# Exercise and Weight Management: The Role of Leptin—A Systematic Review and Update of Clinical Data from 2000–2022

**DOI:** 10.3390/jcm12134490

**Published:** 2023-07-05

**Authors:** Gilmara Gomes de Assis, Eugenia Murawska-Ciałowicz

**Affiliations:** 1 Department of Physiology and Pathology, School of Dentistry, UNESP, São Paulo State University, Araraquara 14801-903, Brazil; 2 Department of Physiology and Biochemistry, Wroclaw University of Health and Sport Sciences, 51-612 Wroclaw, Poland

**Keywords:** leptin, metabolism, appetite, weight loss, feeding disorders

## Abstract

A well-balanced metabolism means a lower risk for metabolism-related neuropsychiatric disorders. Leptin is a secretory adipokine involved in the central control of appetite that appears to play a role in the etiology of feeding-related disorders. Additionally, the influence of exercise on feeding behaviors potentially modulates the circulation of metabolites that signal through the central nervous system. In this systematic review, we collected the recent clinical evidence on the effect of exercise on leptin concentrations in health individuals published from 2000 to 20 September 2022, according to the Preferred Reporting Items for Systematic Review and Meta-Analysis Protocols (PRISMA 2020 statement). Six hundred and thirty-eight papers were retrieved and forty-eight papers were included in the qualitative synthesis. Data supports that exercise positively influences appetite via enhancing peripheral and central leptin signaling (reuptake), especially during weight loss. Exercise modulation of leptin signaling through leptin receptors helps to stabilize increases in food intake during periods of negative energy balance, prior to a decrease in the body fat tissue content. At a high intensity, exercise appears to counteract leptin resistance.

## 1. Introduction

Obesity and its associated comorbidities represent a major public health issue to the economy of developing countries which also show high profiles in sedentary behavior [1], and overweight individuals are likely to have poorer fitness conditions worldwide [2,3]. The positive impact of exercise is seen in various cardiometabolic parameters [4]. Regulation of the adipokine leptin is essential in the control of appetite, as it plays a key role in the prevention of metabolic syndrome-associated dementia [5,6]. The levels of leptin found in circulation are around 7.5 ± 9.3 ng/mL in healthy non-obese individuals, and 31.3 ± 24.1 ng/mL in individuals with obesity [7,8]. Blood leptin levels highly correspond to the body fat content and seem to be fairly stable. This is observable in the study by [9], which exposed lean and obese women to high-fat and high-carbohydrate meals with similar energy content, and found no differences in meal-induced thermogenesis or leptin levels. From another perspective, ref. [10] reported a decrease in the leptin levels of young and lean men that was consistent after two different types of high-fat meals. Conversely, in the study by [11], evidence was found to show that hypocaloric diets with a high-fat profile induce smaller decreases in leptin levels in individuals with obesity, which display high-leptin levels, compared to those induced with diets with a low-fat profile. These evidences suggest that the hormonal action of leptin might exert a feedback communication between the fat metabolism/storage and central nervous system (CNS) circuits related to feeding.

Synthetized in adipocytes, leptin is secreted into the bloodstream and enters the brain either by a saturable transport system at the blood–brain barrier or via cerebrospinal fluid. In the central nervous system (CNS), leptin activates a long-leptin receptor (LLR) isoform, mainly expressed in neurons within the hypothalamic areas of the brain, but is also present on all types of immune cells. Detection of an increase in leptin concentrations by LLR localized in these CNS circuits promotes the feeling of fullness and regulates the stimulus for feeding [12,13,14,15,16,17,18]. Individuals with a higher body mass index (BMI) and greater leptin secretion rates are thus more susceptible to an impairment in the leptin signaling system, which is considered an early risk factor for the development of obesity-related comorbidities and dementia [19,20,21,22].

There is a lot yet to be uncovered about leptin signaling in the brain. The LLR isoform found in central circuits shows a slower recycle rate relative to other leptin receptor (LR) isoforms and a delayed expression on cell membrane surfaces. Animal studies show that impairment in the expression of LLR leads to feeding until obesity; meanwhile, a consistent reduction in leptin levels is required before a loss in body adiposity [23].

In patients with obesity, an excessive circulating leptin is associated with a weaker leptin-LLR signal transduction in the hypothalamus and affect the reward system, characterizing a state of leptin resistance. When faced with misfunctioning in leptin-LLR signaling, higher loads of leptin are required to achieve satiation and feeding self-control [17,24,25,26].

In this context, the regulation of leptin signaling in the brain is of particular importance during the process of weight loss. Nevertheless, while changes in basal leptin levels require a consistent reduction in the energy intake, the leptin action in glucose homeostasis precedes changes in body weight. Exercise, besides promoting a negative energy imbalance, might work as a positive regulator of LRR, aiding in the regulation of appetite and weight loss [4,27,28,29].

Therefore, we hypothesize that exercise is a crucial contributor to sustained reductions in leptin levels. In this systematic review, we aimed to synthesize the knowledge regarding the role of exercise on leptin and weight control. To do so, we compiled data from all the clinical studies addressing exercise interventions and analyzed leptin levels in healthy individuals that were published from 2000 to 2022.

## 2. Materials and Methods

**Figure 1:**
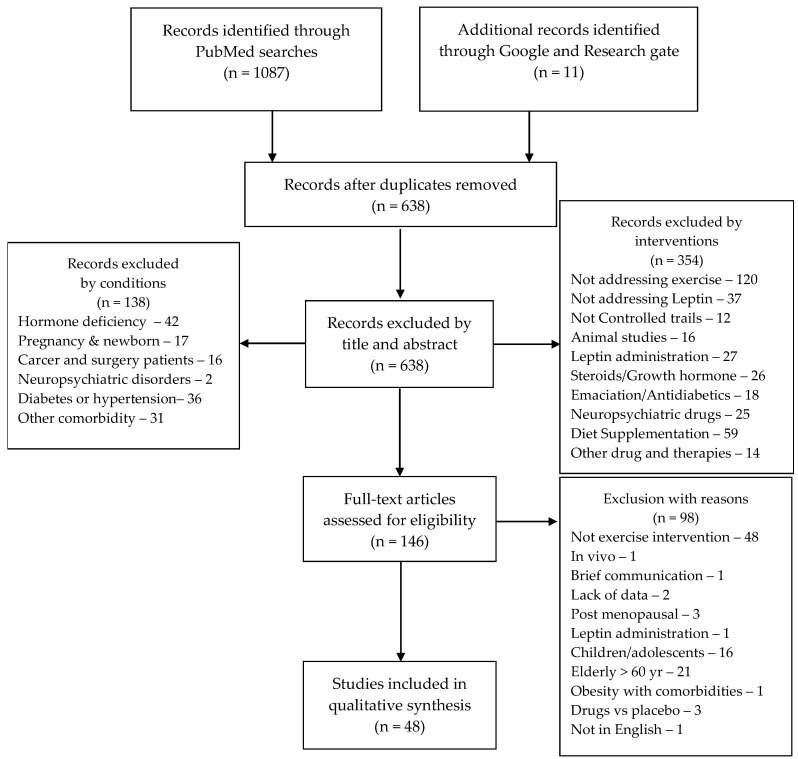
Excessive leptin concentrations on the expression levels of LR (**left**), and the counteractive effect of exercise (**right**) (Created with BioRender.com).

A systematic search of scientific studies addressing exercise interventions was conducted by two independent investigators, in accordance with the Preferred Reporting Items for Systematic Review and Meta-Analysis (PRISMA 2020) statements [30]. To obtain all studies on leptin levels and exercise, versus a control condition, the following Patient, Intervention, Comparison, and Outcome (PICO) strategy was applied to PubMed and Google scholar databases: P—Healthy, active, or sedentary individuals, with or without obesity; I—Sleep and/or diet regimes; C—healthy controls; and O—Leptin and/or leptin receptor. Combinations of these Mesh terms and Boolean operators were used during the searches: “Leptin” *AND* “exercise”, “Leptin” *AND* “Metabolism”; OR “Receptors, Leptin” *AND* “Metabolism”; OR “Leptin” *AND* “exercise”; OR “Receptors, Leptin” *AND* “exercise”. The search filters of *Clinical studies* and *2000 to 2022* publication period were applied. After combining the paper retrieved from the two investigators, duplicates were removed. A total of one thousand and ninety-eight papers were retrieved and six hundred and thirty-eight papers were applicable to include in the discussion after the removal of the duplicates. The retrieved papers dating from 2000 to 20 September 2022, were screened by title and abstract in a double-blinded manner. Papers including SARS-CoV2 or COVID-19 or subjects with comorbidities, other than overweight and obesity, were not considered for review. Overall, one hundred and forty-six full texts were assessed regarding the inclusion criteria of non-randomized and randomized controlled trials presenting data on leptin and/or leptin receptors in plasma, serum, or tissue analyses in subjects aged from 18 to 60 years, with or without obesity, and undergoing exercise interventions. Reviews, cohorts, brief communications, studies involving menopause and pregnancy, drugs and supplementation, as well as those studies addressing populations with associated pathological conditions were excluded. All exercise models (e.g., aerobic exercise, high intensity interval training (HIIT), resistance training) were included. There were no restrictions on intervention duration. Control groups and baseline measurements were comparators for intervention effects. Control groups that included any type of exercise intervention were considered to be intervention groups. Risk of bias items were recorded on a pre-formatted spreadsheet (not published) and the studies’ quality were assessed by two raters using the Cochrane Risk of Bias (Rob 2 tool). Included studies were examined for the adequacy of randomization, allocation concealment, blinding of participants and personnel, blinding of outcome assessment, incomplete outcome data, selective reporting, and other sources of bias, when applicable. Those criteria items were evaluated as a high, low, or unclear risk. A consensus was reached through discussion for all disagreements or misunderstandings by two independent authors (G.G.A. and E.M.-C.). After the quality assessment, forty-eight papers were included in the qualitative analysis (Figure 1).

## 3. Results

The study by Hilton & Loucks [31] examined the effects of energy availability and exercise on diurnal leptin rhythms in non-overweight, young women by controlling energy intake and exercise energy expenditure, and found that diet suppressed the 24-h mean and amplitude of diurnal leptin levels, whereas exercise did not. Zaccaria and colleagues [32] assessed the relationship between energy expenditure and changes in serum leptin concentrations in active men during a long, prolonged, and ultra-long endurance race. Pre-race leptin values decreased during the ultra-long and prolonged races, but not during the long one (half-marathon). The Tsofliou, F. et al. [33] study identified a correlation between serum leptin levels and appetite regulation in response to moderate physical activity in obese women. A summary of the studies data is displayed in Table 1.

The Kraemer, R. R. et al. [34] study, with active men undergoing progressive intensity exercise training, demonstrated that testosterone levels do not acutely affect plasma leptin responses to exercise or 1-hour recovery levels. A varied leptin response to intense exercise was identified in association with baseline leptin concentrations. Data from the Tsai, A. C. et al. [35] study suggested that food restriction is more effective in reducing body weight, while exercise is more effective in reducing body fat and maintaining lean body mass in adult women, consequently improving leptin levels.

Desgorces et al. [36] evaluated the effect of prolonged aerobic exercise on leptin levels in athletic men before and at various time points after two separate rowing exercise sessions early and late in the 8-month training season. Basal leptin levels did not change over the 8 months. A delayed reduction in leptin levels appeared as post-session leptin levels were lower at 120 min and remained lower for 24 h, only for exercise sessions early in the season. The Rubin, M. R. et al. [37] study, with young overweight men undergoing a high-intensity resistance training session, also did not find changes in plasma leptin concentrations.

Kondo et al. [38] studied the effects of 7 months of aerobic exercise on circulating leptin levels in obese young women and the non-obese controls. Exercise resulted in decreased leptin and adiponectin levels in the obese, relative to non-obese participants. Ara et al. [39] examined whether the free leptin index, measured by the molar excess of soluble leptin receptors to leptin, is increased by 6 weeks of resistance training in young men. While fat mass decreased and lean mass increased, serum leptin concentrations were not affected by the training. In contrast, leptin receptors increased by 13% at the end of training while the molar excess of leptin receptors to leptin remained unchanged. The Moro, C. et al. [40] study of sedentary men and women undergoing low- and moderate-exercise sessions reported that exercise did not induce any changes in plasma leptin concentrations. Kyriazis et al. [41] evaluated whether 60 min of moderate-intensity exercise altered leptin levels in obese individuals. However, no effects of exercise were observed in leptin immediately after exercise, 24 h post-exercise, or 48 h post-exercise.

König et al. [42] assessed the alterations in body weight, fat mass, and leptin levels following 6 weeks of increased physical activity along with either a meal-replacement regimen or a low-calorie diet. Leptin and insulin were reduced with both regimes as they lost weight and fat mass. Volpe et al. [43] investigated the effects of diet, exercise, and the combination of diet and exercise on body weight, body composition, energy intake, and leptin levels in sedentary, obese adults. Diet with exercise promoted a greater loss in body weight compared to both diet and exercise alone. Serum leptin levels decreased following 6 months of diet and diet with exercise in women, and with 6 months of exercise in men.

Trapp et al. [44] investigated the effects of a 15-week HIIT program on trunk fat and metabolic parameters in young women. The subjects had decreases in leptin levels that negatively correlated with fitness improvement and positively correlated with decreases in total body mass. Liu et al. [45] evaluated the metabolic effect of detraining in elite kayak athletes subjected to total or partial detraining for one month. Elevations in the areas under the curve for fasting leptin levels were observed with detraining. Changes were greater in the totally detrained participants.

**Table 1:** Clinical studies designs and main findings.

Author and DOI	Subjects and Design	Main Outcomes
Hilton & Loucks [31]. DOI 10.1152/ajpendo.2000.278.1.e43	Young women (age range, 20–22 yr, BMI, 24–26 kg/m^2^) engaged in either an exercise or diet intervention, with controlled energy intake, were evaluated for diurnal leptin levels during the estrous cycle.	Low energy availability suppressed the 24 h mean and amplitude. The negative effects of low energy intake on the leptin rhythm were more pronounced in sedentary women.
Zaccaria, M. et al. [32]. DOI 10.1007/s00421-002-0606-4	Active men (age range, 32–49 yr, BMI, 21–27 kg/m^2^) engaged in either a half-marathon run (21.097 km—1400 kcal), a ski-alpinism race (about 45 km—5000 kcal), and an ultramarathon race (100 km—7000 kcal) were evaluated for energy expenditure and leptin levels.	Serum leptin decreased 3% in the ultramarathoners and 7% in the ski-alpinism athletes but did not change after the half-marathon. Variation in free fatty acids was inversely correlated with variation in leptin.
Tsofliou, F. et al. [33]. DOI 10.1038/sj.ijo.0802406	Adult women (age range 42–58 yr, BMI, 30–37 kg/m^2^) were tested for blood leptin before and after a 20 min brisk walk, a 58.5 g chocolate-based snack, and control (sitting, TV-watching) conditions.	There was no effect of any intervention or effect over time on serum leptin concentrations. No associations were found between serum leptin and blood glucose or plasma free fatty acids.
Kraemer, R. R. et al. [34]. DOI 10.1385/ENDO:21:3:261	Active men (age range, 25–32 yr, BMI, 10–12 kg/m^2^) completed an intermittent treadmill workout at the progressive intensity of 60% for 10 min, 75% for 10 min, 90% for 5 min, and 100% VO2max for 2 min; with samples collected 40 and 10 min prior, and immediately after exercise.	Exercise increased leptin concentrations after 5 min at 90% of VO2max, then declined to resting values during recovery.
Tsai, A. C. et al. [35]. DOI 10.1016/S0955-2863(03)00105-0	Adult women (age range 22–55 yr, BMI, 17.4–40.3 kg/m^2^) underwent a 9-day energy deficit period with either pre-planned diet or activity schedule and a food-repletion period. Those on dieting would consume 75% of baseline energy intake and maintained the level of activity. Those in exercise achieved the same energy deficit by performing extra daily physical activity while maintaining the baseline energy intake. Samples were collected from baseline and after treatments.	Blood leptin concentration similarly reduced after exercise and food restriction.
Desgorces, F. D. et al. [36]. DOI 10.1007/s00421-003-1030-0	Male rowers (age range, 20–23 yr) were evaluated for leptin levels before, 120 min after and 24 h after a 90 min rowing session at 70–75% of VO2max throughout the season.	Resting leptin levels were lower after the program. After the program, the past-task leptin levels were reduced after 120 min of recovery and returned to baseline levels after 24 h of recovery.
Rubin, M. R. et al. [37]. DOI 10.1249/01.MSS.0000155402.93987.C0	Young men (age range, 20–26 yr, BMI, 25–31 kg/m^2^) underwent a resistance training session with the first of six sets at 10 RM (80–85% of the 1 RM) with a 2 min rest between sets, and weight was adjusted to allow completion of that set and subsequent sets.	There was no effect of exercise on plasma leptin concentrations.
Kondo, T. et al. [38]. DOI 10.1507/endocrj.53.189	Young women (age range, 18–23 yr; BMI > 25 kg/m^2^) undergoing a 7-month, moderate aerobic exercise program were tested for leptin, adipokine, among other metabolites.	Leptin levels were higher and adipokine levels were lower in women with obesity compared to controls. Adiponectin levels were negatively correlated with BMI and leptin.
Ara, I. et al. [39]. DOI 10.1017/BJN20061956	Healthy men (age range, 21–25 yr; mean BMI, 24 kg/m^2^) were examined for serum free leptin index in a 6-week resistance training program.	Resistance training did not alter basal leptin levels. Changes in leptin were not related to leptin levels, leptin receptor, or free testosterone.
Moro, C. et al. [40]. DOI 10.1038/oby.2007.267	Sedentary subjects (age range, 26.2–28.4 yr; BMI, 27–28 kg/m^2^) performed two exercise sessions: 1, 30 min cycling at 30% of VO2max followed by 30 min at 50%; and 2, 30 min at 30% followed by 30 min at 70% of VO2max.	Plasma leptin concentrations were higher in women than in men, and exercise did not induce any changes in plasma levels of leptin.
Kyriazis, G. A. et al. [41]. DOI 10.1097/JSM.0b013e31802e9c38	Men with obesity (age range, 22–26; BMI, >30 kg/m^2^) submitted to a 60 min treadmill test at 50–60% of VO2max and controls were evaluated for leptin levels.	No changes were observed for leptin, ghrelin or insulin levels during exercise or following recovery.
König, D. et al. [42]. DOI 10.1159/000119416	Adult men and women (age range, 40–55 yr; BMI, 29–33 kg/m^2^) participated in a 6-week, increasing physical activity and either a meal-replacement or a low-calorie diet.	Leptin and insulin levels were reduced in both groups.
Volpe, S. L. et al. [43]. DOI 10.1080/07315724.2008.10719691	Overweight women and men (age range, 37–52 yr; BMI, 27–33 kg/m^2^) were investigated for the effect of 6-month diet and/or exercise program on leptin levels.	Decreases in leptin levels were observed in the diet, and diet and exercise groups. In the exercise group, a decrease in leptin levels was observed only in males.
Trapp, E. G. et al. [44]. DOI 10.1038/sj.ijo.0803781	Young women (age range 18–22 yr; BMI, 21–25 kg/m^2^) were evaluated for subcutaneous and metabolic parameters pre- post a 15-week, high-intensity intermittent cycling program.	There was a decrease in basal leptin levels after the program.
Liu, T. C. et al. [45]. DOI 10.1080/02640410801885925	Elite kayak athletes (age range, 20–22 yr; BMI, 23–25 kg/m^2^) were examined for leptin levels in a partial detrained vs. total detrained condition.	Both partial and total detraining result in elevated basal leptin levels, while the totally detrained athletes had greater increases in leptin levels than the partially detrained athletes.
Jürimäe, J. et al. [46]. DOI 10.1249/MSS.0b013e31818313e6	Male rowers (age range, 18–22 yr; body fat, 7–14%) were tested for their hormonal response to a 2 h rowing session and follow up.	Leptin levels decreased by 20% during the 30 min follow up. Basal leptin levels correlated with energy expenditure, energy intake and basal testosterone values.
Cheng, MHY et al. [47]. DOI 10.1016/j.appet.2008.09.015	Active men (age range, 19–29; Body fat, 5–13%) were tested for their hormonal response to meal consumption followed by exercise, exercise followed by meal, or meal consumption only.	No effects were observed in plasma leptin levels.
Hagobian, T. A. et al. [48]. DOI 10.1152/ajpregu.90671.2008	Young adults (age range, 16–38 yr; BMI, 22–32 kg/m^2^) were evaluated for sex differences in hormones and appetite in response to four exercise bouts with or without energy imbalance.	Lower leptin levels were associated with lower appetite following exercise with induced energy deficit.
Guadalupe-Grau, A. et al. [49]. DOI 10.1152/japplphysiol.91469.2008	Young men and women (age range, 20–26 yr; BMI, 20–28 kg/m^2^) were examined for a sex dimorphism in serum osteocalcin and leptin responses to a 9-week resistance, plus plyometric, training program.	Serum leptin levels correlated with the percentage of muscle phenotype, IIx (myosin heavy chain composition), in men. Serum leptin concentration was reduced with training in women. Changes in leptin levels were associated with fat mass in men but not in women.
Bergouignan, A. et al. [50]. DOI 10.1210/jc.2009-1005	Inactive women (age range, 30–35 yr; BMI, 18–22 kg/m^2^) were evaluated pre-post to the following conditions: a strict 60-day bed rest, or combined aerobic/resistive exercise training concomitantly to bed rest.	No changes in plasma leptin levels were found.
Sartor, F. et al. [51]. DOI 10.1007/s00421-010-1571-y	Healthy adults (age range, 27–47 yr; BMI, 28–36 kg/m^2^) were engaged to a 75% energy expenditure reduction in calorie intake or diet, plus a 2-week HIIT with dietary intake increased via the activity correction factor to compensate for energetic costs.	Plasma leptin was reduced in both groups.
Cooper, J. A. et al. [52]. DOI 10.1016/j.appet.2010.10.009	Healthy men (age range, 18–45 yr; BMI, 18–30 kg/m^2^) were crossed over a 3-day, high-saturated fat or high-monounsaturated fat diet with a cycling session at 45% of VO2max to reach the 24 h EE of 1.8 × resting metabolic rate; and the corresponding sedentary conditions.	Average 24 h leptin levels were lower during exercise vs. sedentary conditions.
Kraemer, R. R. et al. [53]. DOI 10.1249/MSS.0b013e3182114ab9	Healthy men (age range, 20–25 yr; BMI, 20–28 kg/m^2^) performed 90 min of treadmill exercise at 60% of VO2max with blood samples collected twice before, every 18 min during exercise, and every 20 min during 1 h of recovery.	Plasma leptin concentrations remained stable across the measurements.
Moran, C. N. et al. [54]. DOI 10.1016/j.metabol.2009.12.026	Healthy women (age range 28–42 yr; BMI, 23–33 kg/m^2^) with and without a family history of diabetes accomplished a 7-week exercise session performed at 65–80% of maximum HR, with pre-post blood and abdominal subcutaneous adipose tissue samples analyzed.	Leptin mRNA decreased similarly in both groups. Plasma leptin decreased more in those with family history. The expression of the long leptin receptor mRNA increased only in those with family history. Changes in plasma leptin and leptin mRNA correlated with changes in insulin sensitivity.
Balaguera-Cortes, L. et al. [55]. DOI 10.1139/H11-121	Healthy men (age range, 19–23 yr; BMI, 21–26 kg/m^2^) completed 3 trials as: 45 min of resistance exercise, running, or resting control, followed by a breakfast ad libitum.	There was no effect showing exercise effects on pre-prandial plasma concentrations of leptin. There was a time effect, however, with lower leptin concentrations observed post-meal compared with baseline.
Rosa, G. et al. [56]. DOI 10.14310/horm.2002.1311	Adults (age range, 22–32 yr; BMI, 22–28 kg/m^2^) participated in three trail sessions: control, indoor cycling class followed by strength training, and resistance training with a 5-day rest in between, and blood samples were collected in each session.	There was a similar reduction in leptin levels after both exercise sessions.
Arikawa, A. Y. et al. [57]. DOI 10.1249/MSS.0b013e3182059eda	Inactive women (age range, 18–30 yr; BMI, 18–40 kg/m^2^) were evaluated for changes in inflammatory markers during a 16-week aerobic exercise program.	There was no effect of exercise on the levels of leptin or adiponectin.
Kelly, K. R. et al. [58]. DOI 10.1249/MSS.0b013e318228bf85	Adults with obesity (age range, 52–58 yr; BMI, 32–35 kg/m^2^) were tested for adiponectin and leptin after a 7-day, high-intensity exercise program.	There were increases in adiponectin and the ratio of adiponectin to leptin, and a decrease in leptin after the program.
Numao, S. et al. [59]. DOI 10.1159/000346205	Obese males (age range, 46–50 yr; BMI, 28–30 kg/m^2^) were tested for circulating leptin before and after a 12-week exercise training program.	There was a decrease in circulating leptin levels after training.
Zaccaria, M. et al. [60]. DOI 10.3275/8656	Young men (age range, 22–32 yr; BMI, 20–25 kg/m^2^) were assessed for leptin levels during a 4 h treadmill running task.	Plasma leptin levels decreased within 2 h after the task. There were negative correlations between leptin, norepinephrine and energy expenditure.
Martins, C. et al. [61]. DOI 10.1249/MSS.0b013e31827d1618	Sedentary adults (age range, 28–35 yr; BMI, 28–34 kg/m^2^) underwent a 12-week program: 5-day treadmill walking or running at 75% HRmax, for 500-kcal energy deficit per session, per week.	There was a reduction in the fasting and postprandial leptin concentrations after exercise intervention.
Morishima, T. et al. [62]. DOI 10.1111/cpf.12069	Sedentary adults (age range, 28–35 yr; BMI, 24–27 kg/m^2^) accomplished 4-week training 3 days per week at 55% HRmax, under hypoxic and normoxic conditions.	A reduction of postprandial leptin response was observed in both groups.
Mendham, A. E. et al. [63]. DOI 10.1007/s00421-014-2953-3	Sedentary men (age range, 42–54; BMI, 24–31 kg/m^2^) were tested before and after an 8-week cycling, small-sided game training of 3 days/week, and under control conditions.	Both exercise conditions decreased the concentration of plasma leptin.
Debevec, T. et al. [64]. DOI 10.1371/journal.pone.0098874	Healthy men (age range, 21–28 yr; BMI, 19–25 kg/m^2^) underwent a 10-day hypoxic confinement at 4000 m with daily moderate intensity exercise or control conditions.	No differences between groups or testing periods were noticed for plasma leptin concentrations.
Ahmadizad, S. et al. [65]. DOI 10.1016/j.clinbiochem.2013.12.019	Sedentary men (age range, 22–24 yr; BMI, 25–30 kg/m^2^) were allocated to undergo an 8-week resistance training with different protocols of periodization.	No changes were observed in plasma leptin concentrations.
Sim, A. Y. et al. [66]. DOI 10.1249/MSS.0000000000000687	Inactive men (age range, 24–39 yr; BMI, 25–29 kg/m^2^) were randomized into either HIIT or moderate-intensity continuous exercise training at three sessions per week, during 12 weeks.	Leptin concentrations were lower after HIIT but not after moderate control conditions.
Kim, Y. S. et al. [67]. DOI 10.1111/cen.12601	Young men (age range, 22–29 yr; BMI, 26–32 kg/m^2^) were examined for changes in adipocytokines following an 8-week treadmill running exercise at 65–75% VO2max (~600 Kcal), four times per week.	Leptin levels decreased after program. Adiponectin over total adiponectin ratio increased.
King, J. A. et al. [68]. DOI 10.1016/j.physbeh.2014.12.050	Young men (age range, 20–24 yr; BMI, 20–25 kg/m^2^) were investigated for corrective changes in appetite-regulatory parameters on the day after a single bout of exercise.	Circulating leptin levels were reduced on the day after exercise. The AUC for plasma leptin showed reduced circulating levels the day after.
Douglas, J. A. et al. [69]. DOI 10.1016/j.appet.2015.05.006	Young men (age range, 18–23 yr; BMI, 21–25 kg/m^2^) were examined for appetite, food intake and regulatory hormones in response to a single exercise bout.	Fasting leptin levels did not differ following the single exercise bout.
Racil, G. et al. [70]. DOI 10.1139/apnm-2015-0384	Young women (age range, 15–18 yr; BMI, >25 kg/m^2^) were evaluated for biochemical parameters pre-post 2 HIIT programs (with and without plyometrics).	There was a decrease in leptin and an increase in adiponectin levels after both training programs. The plasma leptin/adiponectin ratio was reduced in both programs, but changes were greater in the plyometric + HIIT group.
Kong, Z. et al. [71]. DOI 10.1155/2016/4073618	Young men and women (age ranger, 19–21 yr; BMI, 24–28 kg/m^2^) were analyzed for hormonal responses to a 5-week HIIT compared to moderate-intensity training.	No changes were observed in leptin levels among women.
Cameron, J. D. et al. [72]. DOI 10.3945/ajcn.115.115584	Young men (age range, 18–28 yr; body weight, 71–95 kg) participated in 2 experimental conditions: 25% daily needs energy deficits induced by diet only and by exercise only, and tested before and after 3 and 4 days of the intervention.	There was no condition effect for plasma leptin concentrations.
Vardar, S. A. et al. [73]. DOI 10.1080/13813455.2017.1369998	Young women (age range, 18–25 yr; BMI, 25–35 kg/m^2^) completed 19 days of high-intensity training through six sessions of 4–6 repeats of a Wingate test. Plasma adipokine levels were measured before exercise, and at 5 and 90 min after exercise, on the first and last training days.	Plasma leptin concentrations decreased 5 min after exercise and remained reduced following 90 min in both the first and last training days.
Caldeira, R. S. et al. [74]. DOI 10.1016/j.clnesp.2018.08.005	Twenty men (are range, 18–35 yr; BMI, 18–29 kg/m^2^) performed 5 weeks of HIIT (5 km: 1 min running at 100% speed/1 min passive recovery), or running 5 km at 70% of the VO2max, 3 days a week. Leptin was analyzed at baseline, 24 h, and 48 h after an exercise session pre- and post-five weeks.	Plasma leptin concentrations were reduced in both exercise groups. LR increased post-five weeks of HIIT but continuous intensity exercise training.
Tremblay, A. et al. [75]. DOI 10.1139/apnm-2019-0019	Older adult men and women (age range, 50–70 yr; BMI, 25–35 kg/m^2^) were tested for plasma leptin and ghrelin levels responses to a 12-month, high-resistance and low-aerobic exercise, low-resistance and high-aerobic exercise, and low-resistance and low-aerobic exercise.	Leptin levels decreased during the first 3 months and then plateaued.
Sommer, C. et al. [76]. DOI 10.1210/jc.2017-02126	Adult men (age range, 40–65 yr; BMI, 21–27 kg/m^2^) were analyzed for circulating levels of leptin receptors (LR) and related genes, as well as mRNA pathways in adipose tissue and skeletal muscle pre-post a 12-week intensive exercise program.	Plasma LR levels were correlated with leptin, fasting GLU and insulin sensitivity. High adipocyte LR expression was associated with the upregulation of oxidative phosphorylation, adipogenesis, fatty acid metabolism and peroxisomes, and downregulation of inflammatory response. In muscle, high-LR expression was associated with the upregulation of the pathways of oxidative phosphorylation, and KRAS and mTORC1 signaling, and downregulation of an inflammatory response.
Middelbeek, R. J. W. et al. [77]. DOI 10.1038/s41387-020-00144-x	Sedentary men (age range, 40–55 yr; BMI, 18.5–30 kg/m^2^) performed 6 sessions of 6 × 30 s all-out cycle ergometer sprints with 4 min of recovery between sprints, or cycle ergometer exercise at 60% VO2max gradually increasing in duration from 40 to 60 min, over 2 weeks.	Both training programs reduced the concentrations of plasma leptin.
Zaman, G. S. et al. [78]. DOI 10.1155/2021/6684167	Men and women (age range, 30–60 yr; any BMI) were checked for the impact of 12-week resistance exercise on biomedical profile at high altitude.	Leptin and Interleukin-6 decreased, while adiponectin increased.

Jürimäe and colleagues [46] measured the plasma leptin response to a 2 h rowing session—estimated energy expenditure of 1200–1500 kcal—in competitive male rowers. Plasma leptin decreased and ghrelin concentrations increased 30 min post-exercise. Cheng et al. [47] investigated the influence of exercise timing relative to meal consumption on hormone regulators in active young men. Exercise following a meal consumption or exercising before food intake did not influence plasma leptin concentrations.

Hagobian et al. [48] investigated sex differences in hormone regulation and appetite perception in response to exercise. Participants completed four bouts of exercise in energy balance and four bouts of exercise in an energy-deficient state. Leptin concentrations were not different across conditions in either sex. Guadalupe-Grau et al. [49] investigated a possible dimorphism in leptin responses to a 9-week resistance training program combined with plyometric jumps in healthy subjects. Fat mass was not altered but serum leptin was reduced following training in women. The Bergouignan et al. [50] study used a bed-rest model to examine the long-term effects of physical inactivity on energy balance regulation in lean women by comparing the effects of a strict 60-day bed rest regimen to a combined aerobic/resistance training program concomitant to bed rest. Leptin did not change during the bed rest period in either group, and no group effect was observed.

In the Sartor, F. et al. [51] study, both a 75% caloric reduction diet and energy-balanced HIIT reduced levels of plasma leptin in healthy overweight adults. The Cooper, J. A. et al. [52] study, with healthy men submitted to two different high-fat diets plus exercise, showed that the high saturated or unsaturated fat diets did not differ with respect to markers of hunger or satiety, and exercise decreased 24 h leptin.

In the study by Kraemer, R. R. et al. [53], young men performing a 90 min moderate exercise session with plasma leptin levels were analyzed throughout, and no changes were found in plasma leptin concentrations during exercise and recovery time. The Moran, C. N. et al. [54] study of tissue analyses of adult overweight women with and without a family history of diabetes, and undergoing a 2-month exercise program, showed that exercise exerts different effects on leptin-related variables between women with and without a family history of diabetes.

The Balaguera-Cortes, L. et al. [55] study of non-obese men participating in resistance vs. endurance training did not identify an exercise-induced change in plasma leptin levels in either group. The overweight adults undergoing different concurrent training profiles in Rosa, G., Dantas, E. H. M., & de Mello, D. B. [56] displayed similar reductions in serum leptin concentrations.

Arikawa et al. [57] investigated the effects of a 4-month aerobic exercise program on inflammatory markers in sedentary women. No effects of exercise on adiponectin or leptin were found. Neither a change in body fat percentage or change in fitness influenced the effects of exercise on these inflammatory markers. Kelly et al. [58] examined the effects of a 7-day exercise (85% HRmax intensity) program on adiponectin and leptin secretion in obese adults. There was an increase in adiponectin and a decrease in leptin. The increase in adiponectin was positively associated with an increase in basal fat oxidation.

Numao et al. [59] analyzed the effect of a 3-month exercise program on metabolic factors in obese men. The training resulted in a decrease in circulating leptin and IL-6 concentrations. Zaccaria et al. [60] evaluated the effect of exercise duration and EE on leptin levels during a 4 h treadmill running session performed above 65% of VO2max in young, trained males; plasma leptin levels decreased after the second hour. Plasma epinephrine and norepinephrine progressively increased during exercise and were negatively correlated with leptin and energy expenditure. The overweight adults in the Martins, C. et al. [61] study presented a reduction in leptin concentrations after 12 weeks of regular aerobic exercise. It was suggested that exercise improves the accuracy of compensation for previous energy intake, independent of weight loss. Reductions in plasma leptin levels were observed in overweight sedentary adults after 4 weeks of a moderate-to-low intensity exercise program in hypoxic and non-hypoxic conditions in the Morishima, T. et al. [62] study, and after 8 weeks of a moderate intensity exercise program in overweight sedentary men in the Mendham, A. E. et al. [63] study. However, no reductions in leptin levels were found in active lean men undergoing a 10-day moderate-intensity exercise program in hypoxic conditions in the Debevec, T. et al. [64] study. The Ahmadizad, S. et al. [65] study of sedentary overweight men undergoing an 8-week resistance training program, it was identified that short-term resistance training does not influence leptin or adiponectin.

In the Sim, A. Y. et al. [66] study, inactive overweight men had reductions in plasma leptin concentrations during a HIIT program, but not in the one-year moderate continuous exercise program. Kim et al. [67] evaluated the changes in osteocalcin and leptin levels following an 8-week aerobic exercise program in young obese participants. Leptin levels decreased and adiponectin increased after the intervention. There were no correlations between osteocalcin and total adiponectin or leptin levels. King et al. [68] measured appetite-regulating parameters on the day after a 90 min bout of aerobic exercise in young males and detected a reduction in circulating leptin levels. No change was seen in circulating acylated ghrelin, insulin, or appetite perception. Next, Douglas et al. [69] investigated appetite, food intake, and appetite-regulating hormones (acylated ghrelin, total peptide-YY, leptin, and insulin) over 2 days of 60 min aerobic exercises in young males. No effects were detected on acylated ghrelin or leptin levels.

Racil et al. [70] compared the effects of a 12-week high-intensity interval exercise program versus plyometric exercises (squat jump) and high-intensity interval exercise on metabolic parameters in young obese females. The intervention with plyometrics induced a greater increase in lean body mass and plasma adiponectin concentration, and a decrease in plasma glucose and leptin concentrations. Kong et al. [71] compared the effects of 5 weeks of HIIT versus moderate-intensity continuous training on cardiorespiratory and metabolic parameters in overweight and obese young women. No changes in serum levels of testosterone, cortisol, growth hormone (GH), or leptin were detected.

The Cameron, J. D. et al. [72] study showed that a 4-day diet or exercise program had no effects on leptin levels in young overweight men, regardless of the changes observed in appetite. Overweight women in the Vardar, S. A. et al. [73] study showed decreases in plasma leptin concentrations after 19 days of HIIT and the changes in adiponectin and leptin concentrations were similar on the first and last exercise days. In the Caldeira, R. S. et al. [74] study, young overweight men showed reductions in plasma leptin levels after both HIIT and moderate-to-high intensity continuous training performed for 5 weeks. Only HIIT was able to increase soluble LR levels.

Tremblay et al. [75] evaluated body composition and plasma leptin concentrations in overweight adults undergoing one of three exercise interventions with varying intensities of resistance and endurance over a year. Leptin was lower at day 21 and month 3 and remained stable for the remainder of the 12 months in all groups. Body weight and fat reached a plateau between months 6 and 12. Sommer et al. [76] submitted adult men to a 12-week high-intensity training program and analyzed plasma leptin receptors, candidate genes, and pathways in adipose tissue and skeletal muscle. The level of plasma leptin receptors positively correlated with the glucose infusion rate, which increases with exercise independent of plasma leptin. High-plasma leptin receptors correlated with the upregulation of metabolic pathways and downregulation of inflammatory pathways in both adipose and muscle tissues. The sedentary overweight men in the Middelbeek, R. J. W. et al. [77] study had reductions in plasma leptin concentrations within 2 weeks of either a HIIT or a progressive-intensity exercise training program. Zaman et al. [78] examined selected biochemical markers in obese adults engaged in 12-week resistance training programs and reported that leptin decreased, while adiponectin increased with exercise.

## 4. Discussion

The circulating levels of leptin decrease under the conditions of fasting, decreasing thermogenesis, and increasing secretion of stress steroids. Meanwhile, increases in leptin signaling via LRs in hypothalamic anorexigenic neurons exert an anorectic effect via secretion of neuropeptides, such as Neuropeptide Y and peptide YY, though the mechanisms underlying these effects have not been fully explained. The studies show that the energy deficit due to training reduces the body fat mass and improves the anorexic effect of leptin signaling in the hypothalamus. The effect of exercise on leptin secretion thus influences weight loss by stabilizing the appetite during periods of negative energy imbalances, such as in long-term diets [48,55,68].

The results of [48,50] suggest that a reduction in basal levels of leptin are more related to changes in the appetite than with exercise demands, and did not occur after an exercise program based the energy consumption of 30% of total daily energy expenditure, but were associated with food intake and appetite. An effect of exercise on leptin signaling, however, might be associated with the metabolic demands imposed by exercise. When faced with an increase in the metabolic rate, adipocytes secretion of leptin gradually rise and leptin signaling via LR in skeletal muscle induces the expression of peroxisome proliferator-activated receptor gamma coactivator 1-alpha (PGC1α) downstream regulators of cell metabolism, which includes the expression of leptin receptors [75,76]. So, in physiological conditions, an increase in leptin concentrations will induce the expression of LRs and leptin reuptake as a positive feedback that favors the leptin signaling [77,78].

Likewise, there appears to be an intensity-dependent relation between exercise and the adaptive responses of leptin signaling and/or leptin reuptake. Data from the reviewed studies report that there was no change in leptin levels in individuals who performed exercise at a low-intensity (i.e., a single exercise session below 60% of VO2max) or in overweight and lean men and women in studies by Kraemer et al., 2011 [53], Kyriazis et al., 2007 [41], Moro et al., 2007 [40], Cheng et al., 2009 [47], or also in the obese women in the Tsofliou et al. [33] study and the young overweight women in the Kondo et al. [38] study. Meanwhile, an acute decrease in leptin levels was found in all obese and non-obese individuals who exercised at an intensity above 70% of VO2max throughout the studies [32,34,51,60,61,66,73,79,80].

Moreover, the study by Sommer et al. [76] demonstrated that plasma concentrations of LRs are upregulated in response to an exercise-induced increase in the glucose infusion rate, independently of leptin concentrations. Such regulation was associated with increased metabolism and decreased with inflammation in the adipose tissue and skeletal muscle. This elucidates a role of leptin in glucose homeostasis via regulation of LR which is modulated by exercise prior to changes in the body weight [23]. Similarly, an increase in the plasma LRs and metabolic factors occurred only in the physically active individuals who exercised at a high-intensity interval training, but not in steady intensity training, in the Caldeira et al. [74] study.

Further, a trend for an improvement in leptin reuptake was found in non-obese men after a single session of resistance training in the Ara et al. [39] study; and changes in the subject’s serum concentrations of leptin and LR were correlated. Resistance training does not obligatory demand an increase in the oxidative metabolism and may not rapidly modulate the same intracellular cascades and gene expression responsive to oxidative stress. In line with this, there were no changes in leptin concentrations in the young overweight men submitted to one session of resistance training [37,55] or two-month resistance training programs [39,65].

In general, exercise training programs with a duration between 4 and 12 months, with a standard frequency of 3 to 4 sessions per week, and/or at intensities above 70% of aerobic capability, were effective in reducing basal levels of leptin, especially in individuals with a higher BMI, in which the intensity becomes a determinant factor [57,71,75,78,81]. Individuals with higher rates of leptin secretion exhibit greater changes in response to weight loss.

Recently, a role for the secretory adipocyte adiponectin on the energy intake regulation has been elucidated. With higher concentration of leptin per adiponectin being associated with metabolic disorders and obesity [82,83,84], adiponectin is seen to play a key role in energy intake regulation by stimulating appetite via mechanisms antagonistic to those of leptin. Some of the studies’ data reported that there was an increase in the ratios of circulating adiponectin per leptin and resting metabolic rate that accompanies both short- or long-term training programs [38,44,49,67].

As previously discussed elsewhere [85], the complexity of leptin actions and its differential interactions with other metabolic factors in the body and in the brain is still a barrier for the development of anorexigenic drugs addressing the leptin system. Taking into the account that leptin signaling via LRs in the reward system regulates energy intake, excessive leptin loads downregulate LRs, thus resulting in feeding behavior [86,87,88,89]. Exercise shows to be crucial for a consistent change in body composition during the process of weight loss, mainly by promoting leptin resensitization prior the reductions in the adipose tissue content and leptin secretion levels [31].

## 5. Limitations

Regarding that there are different metabolic factors regulating feeding behavior, future studies investigating feeding-related behaviors and lipid metabolism should consider including other secretory peptides, such as adiponectin.

## 6. Conclusions

Exercise functions as an appetite modulator during the process of weight loss by improving leptin signaling (LR resensitization) in the reward circuits related to appetite and ameliorating glucose metabolism across tissues. Consistent reduction in the fat mass and, consequently, in blood leptin levels, should be more effective with application of exercise at a moderate to high intensity.

## Data Availability

Not applicable.
